# Supplementary material for: Protease-Sensitive and -Resistant Forms of Human and Murine Alpha-Synucleins in Distinct Brain Regions of Transgenic Mice (M83) Expressing the Human Mutated A53T Protein
Source: Biomolecules. 2023 Dec 13;13(12):1788. doi: 10.3390/biom13121788 (PMC10741842; doi:10.3390/biom13121788)
Supplement: Supplementary file 1 [file biomolecules-13-01788-s001.zip › biomolecules-2661157-supplementary.pdf]

Supplementary information

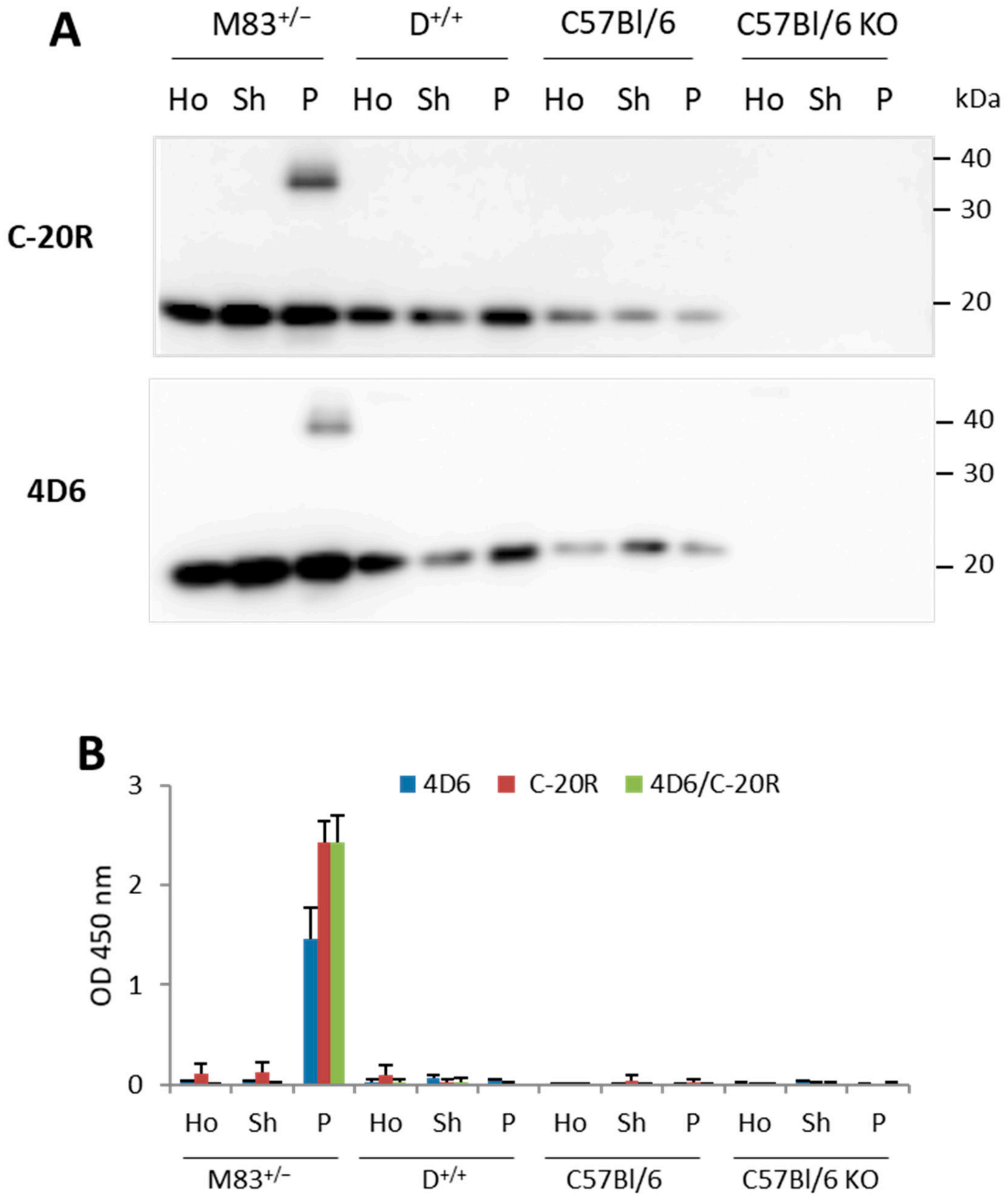

Figure S1. PMCA of M83 brain homogenates induces aS aggregation. (**A**) Western blot with C-20R (against 100-140 aS residues) and 4D6 (against 124-134 aS residues) antibodies reveal SDS-resistant aS aggregates specifically in $M83^{+/-}$ mouse brain , but not $D^{+/+}$, C57Bl/6 and C57Bl/6-KO mouse brains after 144 cycles of PMCA (P), but not after only shaking at 1000 rpm (Sh) or without any treatment (Ho for homogenates). (**B**) ELISA immunoreactivities after loading the same samples on MaxiSorp plates, followed by aS detection using either C-20R or 4D6 antibodies, or using a sandwich ELISA with 4D6 antibody in capture prior to detection by C-20R antibody.

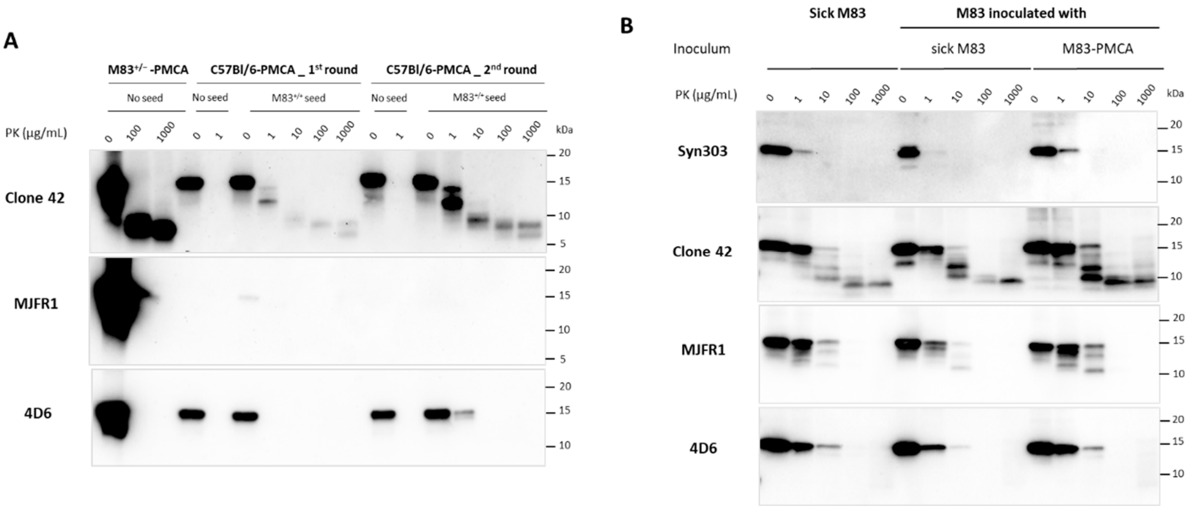

Figure S2. Western blot analysis of aS after PK digestion from 1 to 1000 µg/mL final concentration of PK, in M83$^{+/-}$-PMCA and C57Bl/6-PMCA (M83 seeded) samples (**A**) or M83 brainstem homogenates from i) a sick 16-month old mouse, or two sick M83 mice inoculated with ii) a brain homogenate of a sick M83 mouse or iii) a M83-PMCA sample (**B**) Truncated aS^{res} detected with clone 42 antibody (central) aS region was not identified with the antibodies against the C-terminal aS region (MJFR1 and 4D6) in the PMCA samples and M83 brainstem homogenates, nor with an antibody against the N-terminal aS region (Syn303) in the M83 brainstem homogenates. Bars next to each Western blot panel indicate the molecular weight markers (kDa).

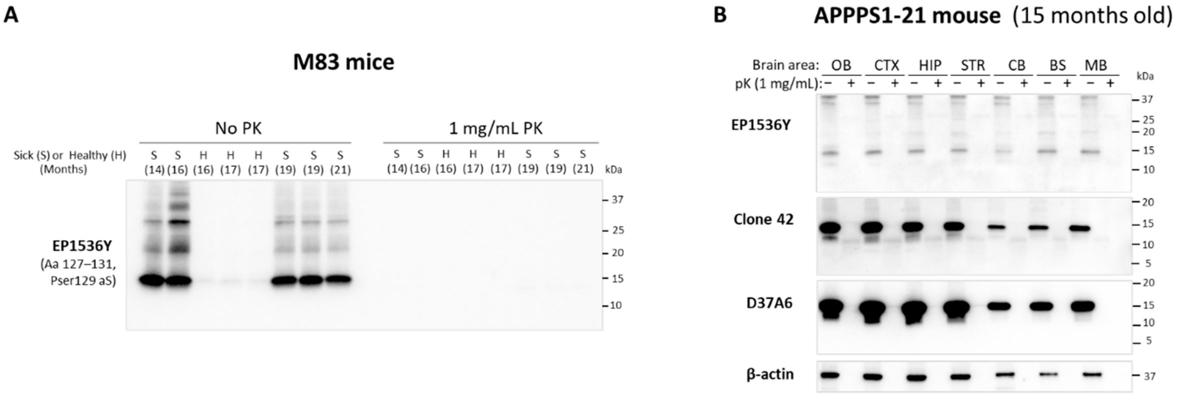

Figure S3. Lack of detection of PK-resistant pSer129-aS in spontaneously sick M83$^{+/+}$ (14-21 months old) described in figure 2, and in APPPS1-21 mice. (**A**) Western blot detection of aS^{res} from M83$^{+/+}$spinal cord samples after digestion with 1 mg/mL of proteinase K and control without PK by E1536Y antibody. (**B**) Samples

from different brain regions were analyzed by Western blotting using antibodies EP1536Y against pSer129-aS, but also clone 42 and D37A6 (murine-specific) against aS, or an antibody against β-actin as a control of protein loads. No truncated forms were detected in any brain region with the three antibodies. Bars to the right of each Western blot panel indicate the molecular weight markers (kDa).

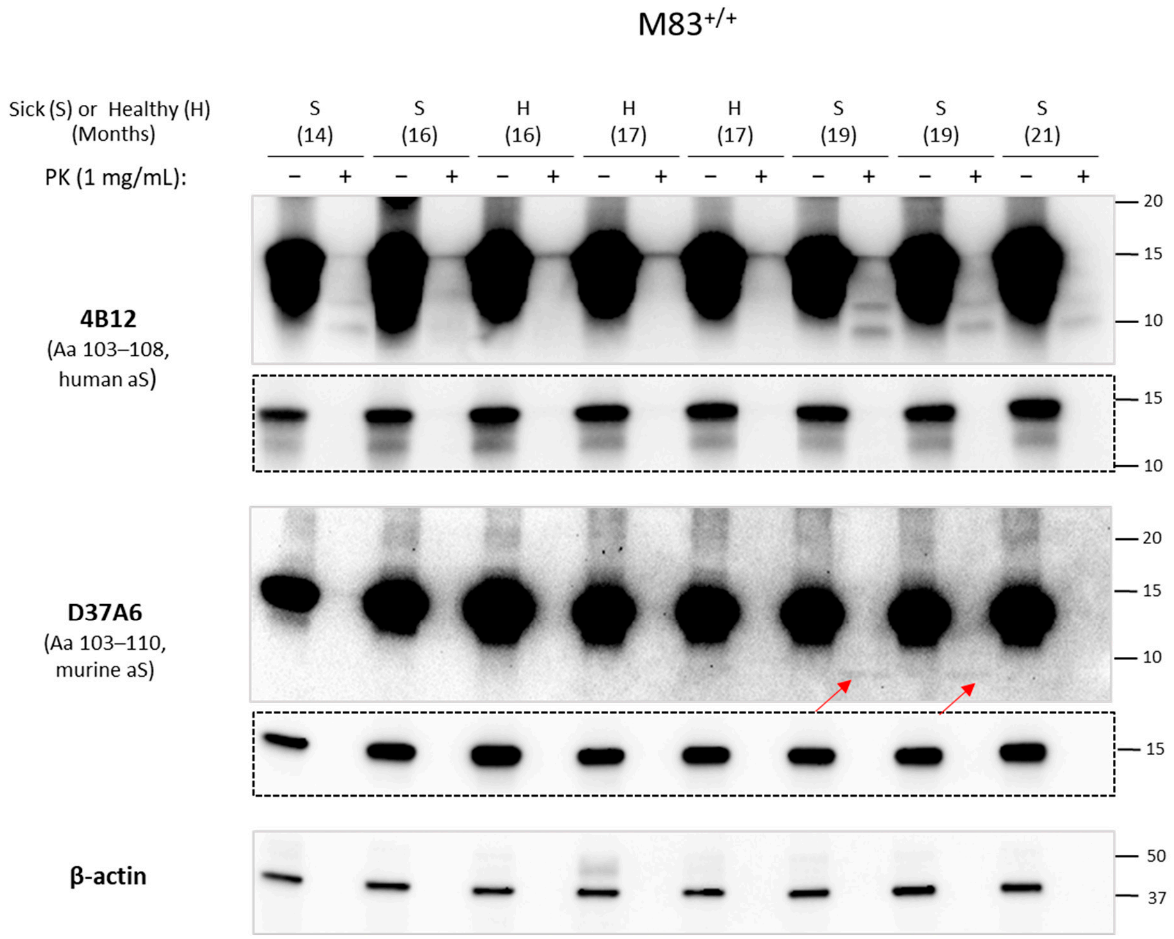

Figure S4. PK resistance of human and murine aS in spontaneously sick M83$^{+/+}$ (14-21 month old) described in figure 2. Western blot detection of aS^{res} from M83$^{+/+}$spinal cord samples after digestion with 1 mg/mL of proteinase K and control without PK by 4B12 (human-specific) or D37A6 (murine specific) antibodies, or an antibody against β-actin as a control of protein loads. Cropped images surrounded by dashed lines from the corresponding condition gel with a shorter exposition are shown below each gel. Red arrows show the presence of murine aS^{res}. Bars next to each Western blot panel indicate the molecular weight markers (kDa).

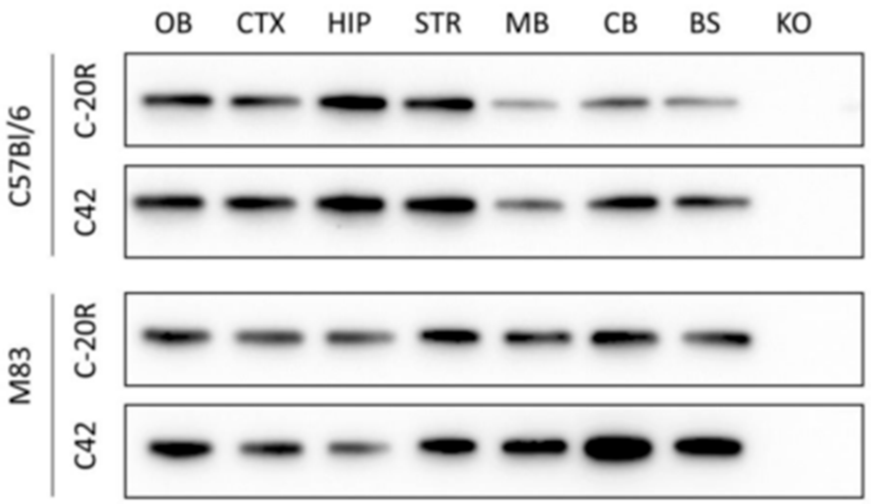

Figure S5: Neuroanatomical distribution of aS in C57Bl/6 wild-type and M83$^{+/+}$ transgenic mice. aS was detected in 7 regions of the brain, with two antibodies C-20R, a C-terminal antibody and clone 42 (C42), recognizing the 91-96 sequence of aS protein. Each frame is positioned at ~18k Da, where aS appears under a monomeric form. C57Bl/6 brains: 200 µg tissue equivalent per lane. M83: 40 µg tissue equivalent per lane. OB: olfactory bulb; CTX: cerebral cortex; HIP: hippocampus; STR: striatum; MB: midbrain; CB: cerebellum; BS: brainstem; SC: spinal cord; KO: sample from the whole brain from mice which do not express murine aS. Bars next to each Western blot panel indicate the molecular weight markers (kDa).

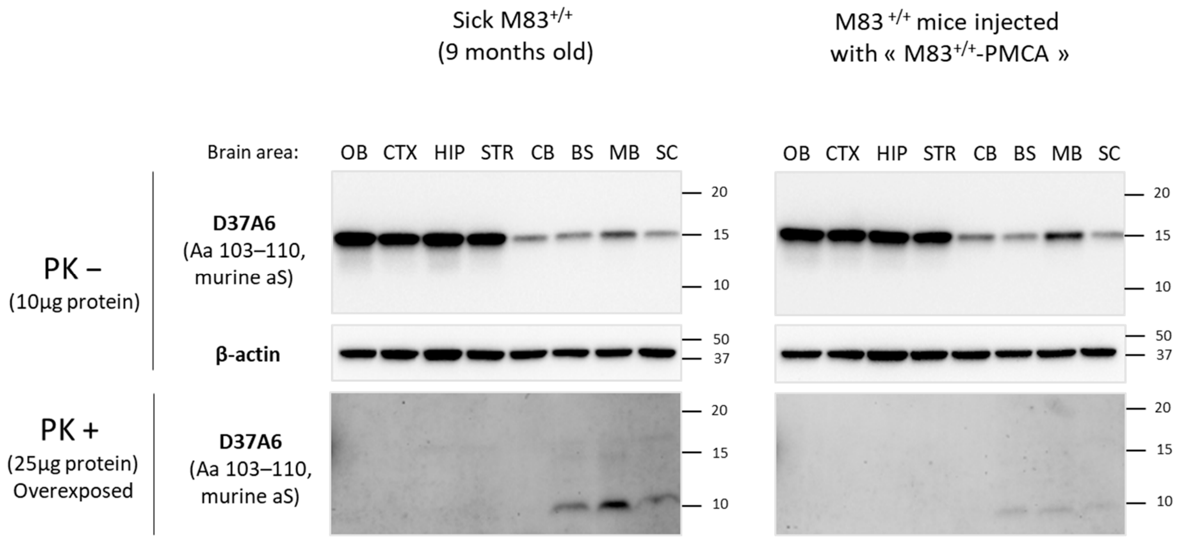

Figure S6: Neuroanatomical distribution of murine total aS and murine PK-resistant aS (aS^{res}) in spontaneously sick M83$^{+/+}$, or M83$^{+/+}$ mice inoculated with M83-PMCA samples. Western blot detection of murine total aS (10 µg protein deposit) without PK digestion (upper panel), or aS^{res} (25 µg protein deposit) (lower panel) after digestion with 1 mg/mL of proteinase K at 37°C for 30 min of different brain areas and spinal cord using D37A6 (murine-specific) or an antibody against β-actin as a control of protein loads. The spontaneously sick M83$^{+/+}$mouse and M83$^{+/+}$ mouse inoculated with M83-PMCA samples showed murine PK-resistant aS (aS^{res}) in midbrain, brain stem and spinal cord, not previously described at lower protein deposition (10 µg) in figure 3A and 6A respectively. OB: olfactory bulb; CTX: cerebral cortex; HIP: hippocampus; STR: striatum; CB: cerebellum; MB: midbrain; BS: brainstem and SC: spinal cord. Bars next to each Western blot panel indicate the molecular weight markers (kDa).

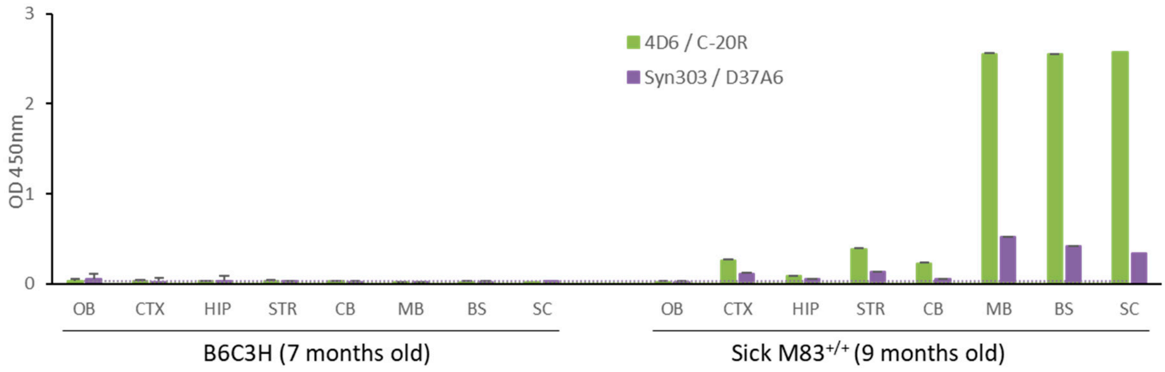

Figure S7. Absence of detectable ELISA immunoreactivity in brain regions and spinal cord of B6C3H mice representing the genetic background of M83 transgenic mice**.** 7 month old B6C3H mice (n = 3) and a M83$^{+/+}$ sick mouse (9 month old) were sacrificed and homogenates were prepared from 7 brain regions and from the spinal cord. Levels of aS immunoreactivities measured using the 4D6/C-20R and Syn303/D37A6 ELISAs showed low immunoreactivities in B6C3H mice, in comparison with that observed, mainly in the midbrain/brainstem and spinal cord, in the M83$^{+/+}$ sick mouse control. Thresholds were calculated as the average of three repeated ELISA tests on four negative spinal cords from young, asymptomatic M83 mice of 1 and 2 months of age, plus three times the SD for each ELISA test. Three replicate ELISAs were performed for each sample. OB: olfactory bulb; CTX: cerebral cortex; HIP: hippocampus; STR: striatum; CB: cerebellum; MB: midbrain; BS: brainstem and SC: spinal cord.

Table S1. Antibodies used

Antibodies	*Epitopic specificity*	*Type*	*Source*	*Dilution (ELISA/WB/IHC)*	*Reference*
Syn303 (α-syn)	2–4	Mouse monoclonal	Biolegend (ref 824301)	1:1000 ^{E} – 1:2000^{WB}	[6]

clone 42 (α-syn)	91–96	Mouse monoclonal	BD Biosciences (ref 610787)	1:4000 ^{WB}	[32]
4B12 (human α-syn)	103–108	Mouse monoclonal	Biolegend (ref 807801)	1:5000 ^{WB}	[38]
D37A6 (mouse α-syn)*	103–110	Rabbit polyclonal	Cell Signaling (ref 4179)	1:1000 ^{E} – 1:1000^{WB}– 1:200 ^{IHC}	[39]
MJFR1 (human α-syn)	118–123	Rabbit polyclonal	Abcam (ref ab138501)	1:5000 ^{WB}	[40]
4D6 (α-syn)	124–134	Mouse monoclonal	Abcam (ref ab1903)	1:1000 ^{E} – 1:4000^{WB}	[41]
C-20R (α-syn)**	100–140	Rabbit polyclonal	Santa Cruz (ref Sc7011-R)	1:5000 ^{E}	[42]
EP1536Y (phosphorylated α-syn)*	PSer129	Rabbit polyclonal	Abcam (ref ab51253)	1:3000 ^{E} – 1:4000 ^{WB} – 1:300 ^{IHC}	[43]
Actin		Mouse monoclonal	Abcam (ref ab8226)	1:2000 ^{WB}	[44]

*Antibody used in ELISA with monoclonal anti α-syn syn303 as capture. **Antibody used in ELISA with monoclonal anti α-syn 4D6 as capture. E: ELISA, WB: Western blot, IHC: Immuno Histo Chemistry.
